# Nasopharyngeal and Oropharyngeal Colonization by *Staphylococcus aureus* and *Streptococcus pneumoniae* and Prognostic Markers in Children with Sickle Cell Disease from the Northeast of Brazil

**DOI:** 10.3389/fmicb.2017.00217

**Published:** 2017-02-15

**Authors:** Larissa C. Rocha, Magda O. S. Carvalho, Valma M. L. Nascimento, Milena S. dos Santos, Tânia F. Barros, Elisângela V. Adorno, Joice N. Reis, Caroline C. da Guarda, Rayra P. Santiago, Marilda de Souza Gonçalves

**Affiliations:** ^1^Fundação de Hematologia e Hemoterapia da BahiaBahia, Brazil; ^2^Centro de Pesquisa Gonçalo Moniz-FiocruzBahia, Brazil; ^3^Faculdade de Farmácia, Universidade Federal da BahiaBahia, Brazil; ^4^Hospital Universitário Professor Edgard Santos - Universidade Federal da Bahia (HUPES-UFBA)Bahia, Brazil; ^5^Instituto Multidisciplinar em Saúde, Universidade Federal da BahiaBahia, Brazil

**Keywords:** nasopharyngeal, oropharyngeal, serotype, *Staphylococcus aureus*, *Streptococcus pneumoniae*

## Abstract

We investigated the nasopharynx and oropharynx microbiota in sickle cell disease (SCD) to identify the microorganisms, antibiotic sensitivity, prevalent serotypes, and association of with laboratorial markers. Oropharynx/nasopharynx secretions were investigated in 143 SCD children aging 6 months to 17 years. Pathogens were isolated using standard procedures, and laboratorial markers were performed by automated methods. *Staphylococcus aureus* (*S. aureus*) was isolated from nasopharynx and oropharynx of 64 and of 17 SCD children respectively. *Streptococcus pneumoniae* (*S. pneumoniae*) was isolated from the nasopharynx and oropharynx of eight SCD patients. Serotypes of *S. pneumoniae* were 19F, 23F, and 14. All isolates were susceptible to penicillin, and patients whose nasopharynx and oropharynx were colonized by *S. pneumoniae* had high concentrations of aspartate transaminase, alanine transaminase, and ferritin. *S. pneumoniae* isolated were not penicillin-resistant serotypes suggesting that the use of penicillin for prophylaxis and/or treatment of infections is safe. Our finding of colonization and laboratory evaluation in SCD patients suggests that microorganisms are involved in the modulation of chronic inflammatory. The association of colonized microorganisms and laboratorial markers suggest a new approach to these patients follow-up, and additional studies of microorganism colonization and their association with SCD patients' clinical outcome will improve control and prevention strategies.

## Introduction

Infections are the major cause of death in children with sickle cell anemia (SCA) (Williams et al., 2009). Likewise, bacterial infection is the primary cause of death during childhood; infants and children younger than 3 years of age are at risk of mortality and morbidity from sepsis (Iughetti et al., 2016). *Streptococcus pneumoniae* (*S. pneumoniae*) is a genetically variable organism that colonizes the human upper respiratory mucosa coexisting with the normal microbiota and establishing a state of asymptomatic colonization (Cardozo et al., 2006; Mitchell and Mitchell, 2014). *S. pneumoniae* is an epidemiologically important pathogen with a worldwide distribution that causes invasive (i.e., pneumonia, bacteraemia, meningitis, sepsis, and arthritis) and non-invasive diseases (i.e., sinusitis, otitis media, conjunctivitis, bronchitis, and pneumonia) (Bogaert et al., 2004b; World Health Organization, 2007; Liñares et al., 2010). *Staphylococcus aureus* (*S. aureus*) infections have recently increased and represent a public health problem in the United States of America (USA) (Bogaert et al., 2004b; Fridkin et al., 2005; Kuehnert et al., 2006). *S. aureus* causes pneumonia, sepsis and osteo-articular, skin, and soft tissue infections (Gonzalez et al., 2005; Moran et al., 2005; Kuehnert et al., 2006). The growing number of community-acquired infections caused by methicillin-resistant *S. aureus* in children and healthy adults is a major problem (Fridkin et al., 2005; Gonzalez et al., 2005; Kuehnert et al., 2006), particularly in countries such as Brazil, where nasal carriage prevalence (48%) (Braga et al., 2014) is higher than those described in other countries of Latin America (Gardella et al., 2011).

Pneumococcal conjugate vaccine has minimal impact on overall carriage rate due to non-vaccine serotypes replacement, but could influence others bacterial species in the nasopharynx (Shak et al., 2013). It has been described after immunization by the 7-valent pneumococcal vaccine an inverse relationship between nasopharyngeal carriage of vaccine type *S. pneumoniae* and *S. aureus*, as well as an increase of infections, especially acute otitis media (Ferreira et al., 2001; Bogaert et al., 2004a; Moran et al., 2005; Kuehnert et al., 2006). Thus, the colonization of the upper respiratory tract may be an important factor in the occurrence of local or systemic disease among SCD patients.

Studies assessing the prevalence of pneumococcal nasopharyngeal colonization demonstrate that the presence of these bacteria may be influenced by several factors, such as age, genetic background, geographic area, and environmental conditions (Ferreira et al., 2001; Bogaert et al., 2004b). Fonseca et al. (2005) evaluated 98 children with SCD in the Brazilian state of Sao Paulo and found that the nasopharynx was colonized by pneumococcus in 13.3% of children. In the USA, the rate of nasopharyngeal colonization in SCD patients ranges from 5 to 17% (Anglin et al., 1984; Steele et al., 1996). Previous studies have described the exposure of these bacteria to penicillin and the selection of antibiotic resistant strains (Denno et al., 2002). However, previous studies assessing penicillin resistance in children with SCD who regularly use penicillin have reported controversial results. Norris et al. (1996) found that 33% of pneumococcus was penicillin-resistant and that 36% of these strains exhibited high resistance. However, Fonseca et al. (2005) found no strains that exhibited high resistance to penicillin. The aims of the present study were to evaluate the nasopharynx/oropharynx colonization in Brazilian children with SCD, identify the penicillin sensitivity profile among *S. pneumoniae*-colonized patients; describe the most prevalent serotypes causing invasive diseases. Furthermore, we described the association of microorganisms' colonization with biochemical laboratorial markers.

## Materials and methods

This study was conducted with 143 SCD patients, aged from 6 months to 17 years, who were prospectively selected from the outpatients at the pediatric hematology department of the Fundação de Hematologia e Hemoterapia do Estado da Bahia (HEMOBA), which is a reference center for clinical assistance, from March to December 2010. All the involved patients were from Bahia, a state on Northeast Brazil and the majority of patients lived in Salvador (state capital) or metropolitan area. All SCD patients were in a steady state, which was characterized by the absence of acute clinical events, infections or inflammatory episodes, and did not undergo any blood transfusion procedures prior to sampling.

Nasopharyngeal and oropharyngeal swabs samples were collected by a researcher on the day of the interview. Additional, clinical and epidemiological information were collected using questionnaires after interviews with the parents; this information was supplemented with information contained in the medical records, such as age, gender, race, SCD genotype (i.e., HbSS and HbSC), prophylaxis, vaccination with the pneumococcal 7-valent and 23-valent vaccines, and laboratory data (i.e., blood cell count, evidence of haemolysis, and evaluation of inflammation).

Nasopharyngeal material was collected during the patients' routine clinic visits using sterile swabs, which were introduced carefully into the right and left nostrils to a depth equal to two-thirds of the distance between the nose and ear lobe (Norris et al., 1996). After the nasopharynx material was collected, another swab was used to collect oropharynx material. Each swab was subsequently immersed in media for bacterial transport and was sent (at room temperature) to the bacteriology laboratory of the Faculdade de Farmácia at the Universidade Federal da Bahia within 1–2 h of collection.

Collected material was inoculated onto blood agar plates containing 5% sheep blood and was incubated at 36 ± 1°C with a 5–10% CO_2_ atmosphere. The presence of a gray-green zone around the colonies (i.e., alpha-haemolysis) was presumptive of *S. pneumoniae*. Confirmation was obtained using the rapid agglutination test with latex particles and by evaluating the inhibitory zone around Optochin differentiation disks; when necessary, the bile solubility test was also performed. Other bacteria were identified using the Gram method to analyse colony characteristics, such as size, color, shape, growth on selective media, and subsequent analysis of biochemical and metabolic activities.

Evaluation of susceptibility to penicillin was performed using the disk diffusion method with oxacillin (1 μg) according to Clinical and Laboratory Standards Institute guidelines (Clinical Laboratory Standards Institute (CLSI), 2012). When the inhibitory zone was >20 mm, the microorganism sample was considered penicillin-susceptible, and when the inhibitory zone was <19 mm, the microorganism was considered penicillin-resistant. The minimum inhibitory concentration (MIC) for penicillin was determined using the *E*-test in all pneumococcal strains resistant to oxacillin (i.e., halo <19 mm). The MIC for penicillin was interpreted as susceptible when the obtained value was <0.06 mg/mL; intermediate when the obtained value was 0.1–1 mg/mL; and resistant when the obtained value was > 2.0 μg/mL (Liñares et al., 2010). Bacterial resistance was also determined for erythromycin, trimethoprim-sulfamethoxazole, clindamycin, and cefotaxime. Serotyping of the isolated pneumococcal strains was performed using the Quellung Neufeld reaction with antisera produced at the Statem Serum Institute in Copenhagen, Denmark.

Chemical and immunological markers were evaluated by immunochemistry and with the A25 Immunoassay system (Biosystems SA, Costa Brava, Barcelona, Spain), the Access® 2 Immunoassay system X2 (Beckman Coulter, Inc., Fullerton, CA, USA) and the Immage® 800 system (Beckman Coulter, Fullerton, CA, USA). Haematological parameters were quantified using an electronic cell counter (Coulter Corporation, FL, USA), and hemoglobin (Hb) patterns were investigated using high performance liquid chromatography (HPLC) (Bio-Rad Variant-I; Bio-Rad, Hercules, CA, USA).

### Statistical analysis

The distribution of quantitative variables was evaluated using the Kolmogorov-Smirnov test. The Mann-Whitney test and the unpaired Student's *t*-test were used to estimate differences in hematological and chemical markers among steady-state SCD children with and without nasopharynx/oropharynx colonization. Differences were considered significant when the *p*-values were <0.05. The statistical analyses were performed using EPI info 6.04 (Centers for Disease Control & Prevention (CDC), Atlanta, GA, United States) and Graphpad Prism Software 5.01 (San Diego, CA).

Multivariate analyses were performed to estimate the likelihood of having pneumonia and infection as outcome (dependent variable) and a possible interaction with oropharynx colonization, neutrophils count, leukocytes count, platelets count, hemoglobin profile, reticulocytes count, and nasopharynx colonization as independent variables, adjusted for sex and age.

## Ethics statement

All children's guardians agreed to participate in the study after reading the terms of informed consent and signing the consent form. After that, the material was collected. The human subject research board of the Centro de Pesquisas Gonçalo Moniz- Fundação Oswaldo Cruz- Bahia (CPqGM-FIOCRUZ-BA) approved the study (CAAE 0031.0.225.000-06). The study was performed after obtaining written consent and followed the Brazilian standards for the development of research on humans. This work was conducted in accordance with the Helsinki Declaration of 1975, as revised in 1983.

## Results

A total of 143 SCD patients were investigated; 44/143 (30.8%) had SC disease (i.e., HbSC), and 99/143 (69.2%) had sickle cell anemia (SCA). The distribution according to sex was 66/143 (46.1%) female patients and 77/143 (53.9%) male patients. The average age of the SCD patients was 9.25 ± a standard deviation (*SD*) of 4.06 years. Also, 41/143 (28.6%) patients were less than or equal to 5 years old, and 102/143 (71.4%) patients were over 5 years old. Clinical manifestations of SCD patients are presented on Table 1.

**Table 1:** **Clinical manifestations of SCD patients**.

**Clinical event**	**Sickle cell disease patients *N* (%)**	**Sickle cell anemia patients *N* (%)**	**SC disease patients *N* (%)**
Hospitalization	117/143 (81.8)	85/99 (85.8)	32/44 (72.7)
Pneumonia	77/143 (53.8)	65/99 (65.6)	12/44 (27.2)
Splenomegaly	21/143 (14.7)	16/99 (16.2)	5/44 (11.4)
Splenic sequestration	18/143 (12.6)	17/99 (17.2)	1/44 (2.2)
Stroke	8/143 (5.6)	7/99 (7.1)	1/44 (2.2)
Vaso-occlusion	119/143 (83.2)	83/99 (83.8)	36/44 (81.8)
Infection	66/143 (46.1)	48/99 (48.5)	18/44 (40.9)
Gallstones	8/143 (5.6)	7/99 (7.1)	1/44 (2.2)

We identified that 85.3% (122/143) of the patients received the pneumococcal 7-valent conjugate vaccine, through the public health Brazilian system. Approximately 14% (14.7%) (21/143) were not vaccinated or received at least one of the three commonly available doses.

Haematological and chemical markers were evaluated in the SCD patients (Table 2). The oropharynx/nasopharynx culture procedure enabled the collection of various microorganisms with an emphasis on *S. aureus* and *S. pneumoniae*. Pneumococcus was isolated from the nasopharynx/oropharynx in 16 of 143 children representing a colonization rate of 11.2%. One child with pneumococcal colonization was colonized by two different serogroups (Table 3).

**Table 2:** **Distribution of variables associated with lipid, renal, and hepatic metabolism; haemolysis; and inflammation in patients with sickle cell disease (SCD)**.

**Markers**	**SCD Patients (*N*) 143**	**Mean ±*SD***	**25th percentile**	**Median**	**75th percentile**
**HEMATOLOGICAL**					
RBC, millions/mL		3.23 ± 0.97	2.47	2.95	3.99
Hemoglobin, g/dL		8.92 ±2.00	7.40	8.50	10.50
Hematocrit, %		27.60 ± 6.20	22.80	26.35	32.92
MCV, fL		87.52 ± 10.81	79.17	87.80	94.93
MCH, pg		28.35 ± 3.75	25.47	28.70	31.00
MCHC, %		32.36 ± 0.99	31.70	32.30	32.90
Reticulocytes, %		7.62 ± 4.84	3.15	6.95	11.22
Erythroblasts		1.61 ± 2.38	–	1.00	2.00
**HEMOGLOBIN PATTERN**					
Hemoglobin S, %		75.23 ± 17.23	56.25	83.10	90.30
Hemoglobin Fetal, %		7.49 ± 6.19	2.70	5.60	10.93
**WHITE BLOOD CELLS**					
WBC, × 10^9^/L		13081.82 ± 5744.83	9475.00	12400.00	15650.00
Neutrophils, × 10^9^/L		6128.33 ± 3801.37	3720.00	5171.00	7648.50
Monocytes, × 10^9^/L		811.23 ± 483.58	433.50	752.00	1034.25
Eosinophils, × 10^9^/L		839.02 ± 777.93	321.75	619.00	1148.50
Lymphocytes, × 10^9^/L		5063.21 ± 2353.83	3319.25	4776.00	6292.25
**PLATELETS**					
Platelets, × 10^9^/L		405.54 ± 158.25	286.00	385.00	502.75
**HEMOLYSIS**					
LDH, U/L		856.41 ± 501.60	444.50	721.00	1256.00
AST, U/L		28.25 ± 21.20	15.00	24.00	32.00
Total Bilirubin, g/dL		2.75 ± 1.77	1.40	2.40	3.70
Direct Bilirubin, g/dL		0.67 ± 0.48	0.35	0.60	0.90
Indirect Bilirubin, /dL		2.08 ± 1.58	0.90	1.70	2.85
**HEPATIC FUNCTION**					
ALT, U/L		48.43 ± 25.31	30.75	24.00	32.00
Total proteins, g/dL		7.33± 0.84	6.80	7.30	7.90
Albumin, g/dL		4.06 ± 0.68	3.60	4.20	4.40
Globulin, g/dL		3.27 ± 0.78	2.75	3.30	3.80
Ratio Albumin/Globulin		1.34 ± 0.46	1.00	1.30	1.5
**KIDNEY FUNCTION**					
Urea, mg/dL		17.73 ± 6.40	14.00	16.50	20.00
Creatinine, mg/dL		0.47 ± 0.18	0.40	0.50	0.55
**LIPID METABOLISM**					
Total Cholesterol, mg/dL		121.27 ± 26.04	103.00	119.00	135.00
HDL-C, mg/dL		35.55 ± 12.39	27.00	33.00	43.00
LDL-C, mg/dL		64.80 ± 22.13	50.50	61.00	78.00
VLDL-c, g/dL		20.83 ± 10.02	14.00	18.00	24.00
Triglycerides, g/dL		104.05 ± 50.10	71.75	91.00	118.00
**IRON METABOLISM**					
Ferritin, ng/mL		374.86 ± 320.16	94.90	182.35	399.37
Serum Iron, mcg/dL		122.27 ± 119.39	71.00	10.00	137.75
**INFLAMMATION**					
Antistreptolysin O, UI/mL		282.86 ± 191.25	29.15	94.00	200.00
CRP, mg/L		11.80 ± 6.98	1.33	3.73	7.35
AAT, mg/dL		152.36 ± 46.01	124.00	156.00	186.00

*RBC, Red blood cells; MCV, Mean Corpuscular Volume; MCH, Mean Corpuscular Hemoglobin; MCHC, Mean Corpuscular Hemoglobin Concentration; LDH, Lactate Dehydrogenase; AST, Aspartate Transaminase; ALT, Alanine Transaminase; HDL, High Density Lipoprotein Cholesterol; LDL-C, Low density lipoprotein cholesterol; VLDL, Very low density lipoprotein; CRP, C-reactive protein; AAT, Alpha-1 antitrypsin*.

**Table 3:** **Profile of the microorganisms identified in the analysis of specimens isolated from the nasopharynx and oropharynx of patients with sickle cell disease (SCD)**.

	**SCD patients *N* (%)**	**Sickle cell anemia patients *N* (%)**	**SC disease patients *N* (%)**
**MICROORGANISMS ISOLATED FROM NASOPHARYNGEAL SPECIMENS**			
0-Normal Microbiota	69 (48.2)	43 (43.4)	26 (59.1)
1-*Staphylococcus aureus*	64 (44.8)	46 (46.5)	18 (40.9)
2-*Streptococcus pneumoniae*	8 (5.6)	8 (8)	0 (0)
3-*Staphylococcus aureus*	1 (0.7)	1 (1)	0 (0)
*Streptococcus pyogenes*			
4-*Streptococcus pyogenes*	1 (0.7)	1 (1)	0 (0)
Total	143 (100)	99 (100)	44 (100)
**MICROORGANISMS ISOLATED FROM OROPHARYNGEAL SPECIMENS**			
0-Normal Microbiota	91 (63.6)	65 (66)	26 (59)
1-*Streptococcus pyogenes*	20 (14)	13 (13)	7 (16)
2-*Streptococcus pneumoniae*	8 (5.6)	5 (5)	3 (6.8)
3*-Enterobacter gergoviae*	1 (0.7)	1 (1)	0 (0)
4-*Staphylococcus aureus*	17 (11.9)	11 (11)	6 (13.6)
5-*Klebsiella pneumoniae*	2 (1.4)	1 (1)	1 (2.3)
6-*Enterobacter aerogenes*	1 (0.7)	1 (1)	0 (0)
7-*Pseudomonas aeruginosa*	3 (2.1)	2 (2)	1 (2.3)
Total	143 (100)	99 (100)	44 (100)

*SCD, sickle cell disease*.

All children under 6 years of age used prophylactic penicillin and received pneumococcal vaccines according to the expected age. None of the pneumococcal isolates exhibited resistance to penicillin. Serotypes and their antibiotic susceptibility are shown in Table 4.

**Table 4:** **Antibiogram and serotype profiles of *Streptococcus pneumonia* isolated from nasopharyngeal (Naso) and oropharyngeal (Oro) specimens from patients with sickle cell disease**.

**Patients**	**Specimen origin**	**Penicillin (E-test)**	**Cefaloxine (E-test)**	**SMX /TMP**	**Erythromycin**	**Clindamycin**	**Serotype**
1	Naso	S	S	R^***^	S^**^	S	NT^*^
2	Naso	S	S	R	S	S	19F
3	Oro	S	S	R	S	S	19F
4	Oro	S	S	S	S	S	23F
5	Naso	S	S	S	S	S	4
6	Naso	S	S	R	S	S	19A
7	Naso	S	S	R	S	S	14
8	Naso	S	S	R	S	S	14
9	Naso	S	S	R	S	S	14
10	Oro	S	S	R	S	S	12F
11	Oro	S	S	R	S	S	9V
12-1^#^	Oro col 1	S	S	R	S	S	23F
12-2^#^	Oro col 2	S	S	R	S	S	3
13	Naso	S	S	R	S	S	19F
14	Oro	S	S	R	S	S	23F
15	Oro	S	S	S	S	S	23F
16	Oro	S	S	S	S	S	23A

* *NT, not typed*;

** *S, sensitive*;

*** *R, Resistant*;

# *Patient colonized by two serotypes*.

*SMX/TMP, sulfamethoxazole/trimethoprim; Naso, nasopharyngeal; Oro, oropharyngeal*.

**Figure 1:**
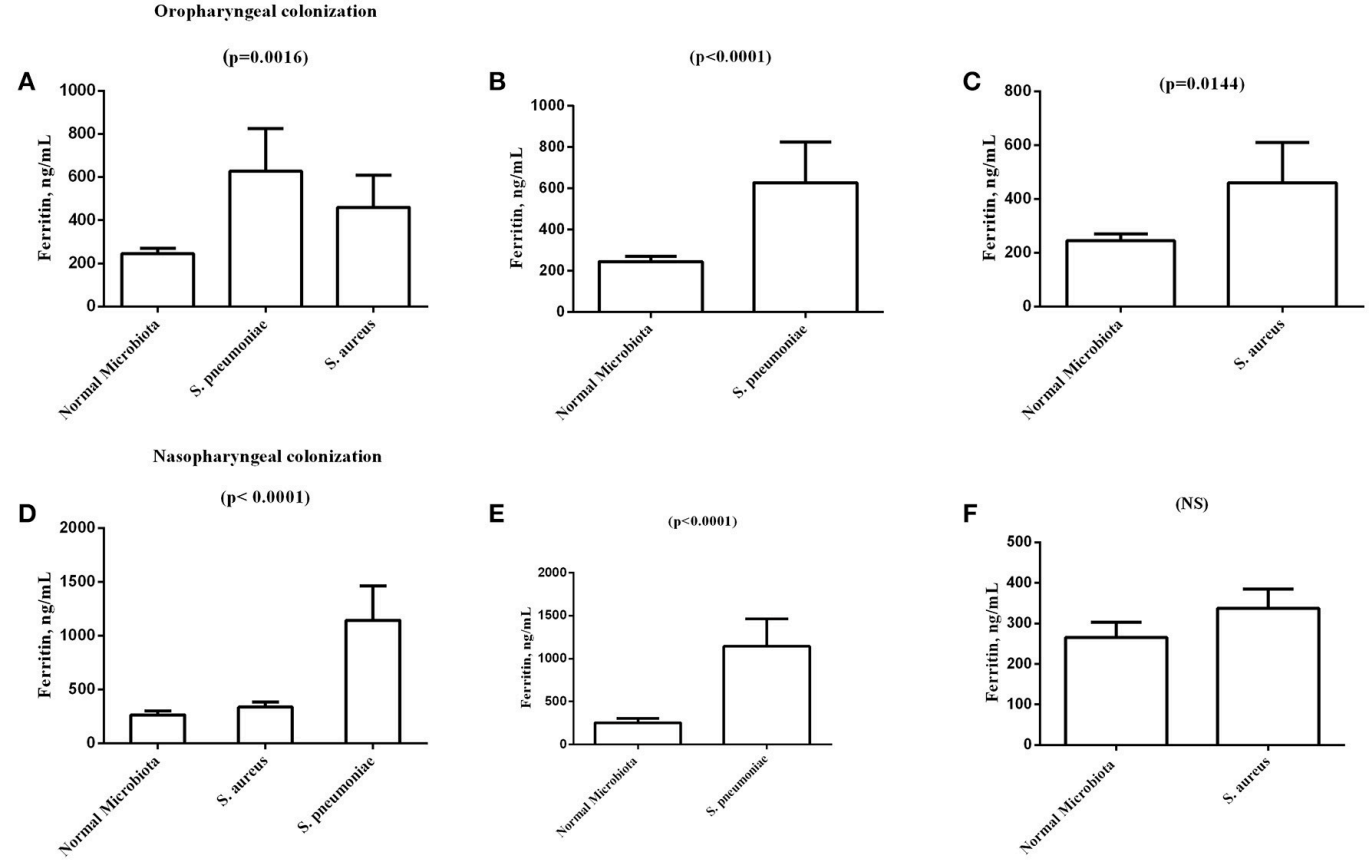
**Graphical representation of the analysis of sickle cell anemia patients' oropharyngeal and nasopharyngeal colonization by normal microbiota, Staphylococcus aureus, and Streptococcus pneumoniae and by its association with ferritin values (ng /mL)**. **(A)** Comparison of ferritin levels among normal Microbiota, *S. pneumonia*, and *S. aureus* in oropharyngeal colonization. **(B)** Comparison of ferritin levels between normal Microbiota and *S. pneumoniae* in oropharyngeal colonization. **(C)** Comparison of ferritin levels between normal Microbiota and *S. aureus* oropharyngeal colonization. **(D)** Comparison of ferritin levels among normal Microbiota, *S. pneumonia*, and *S. aureus* in nasopharyngeal colonization. **(E)** Comparison of ferritin levels between normal Microbiota and *S. pneumoniae* in nasopharyngeal colonization. **(F)** Comparison of ferritin levels between Normal Microbiota and *S. aureus* nasopharyngeal colonization. *S. aureus, Staphylococcus aureus; S. pneumonia, Streptococcus pneumoniae*; NS, Not significant.

An analysis of the association of hematological and chemical markers with nasopharynx and oropharynx colonization revealed a significant association between ferritin (ng/mL) and oropharyngeal colonization by *S. aureus* and *S. pneumoniae* with a mean and SD of 245.1 ± 240.8 for subjects colonized by normal microbiota (91/143); 627.6 ± 624.4 for individuals colonized by *S. pneumoniae* (8/143); and 460.6 ± 148.8 for individuals colonized by *S. aureus* (17/143) (*p* = 0.0016). The analysis also revealed significant differences in ferritin between individuals colonized by normal microbiota and individuals colonized by *S. aureus* (*p* = 0.0144) and between individuals colonized by normal microbiota and individuals colonized by *S. pneumoniae* (*p* < 0.0001) (Figure 1).

A statistical analysis of the association between biochemical markers and the presence of nasopharyngeal colonization by *S. aureus* and *S. pneumoniae* revealed significant differences in ferritin with a mean and standard deviation of 324.0 ± 46.06 for subjects who were colonized by normal microbiota (69/143), 383.1 ± 336.8 for individuals colonized by *S. aureus* (64/143), and 1144 ± 641.8 for individuals colonized by *S. pneumoniae* (8/143) (*p* < 0.0001). The analysis also revealed significant differences when comparing ferritin values in individuals colonized by normal microbiota and individuals colonized by *S. pneumoniae* (*p* < 0.0001) (Figure 1).

**Figure 2:**
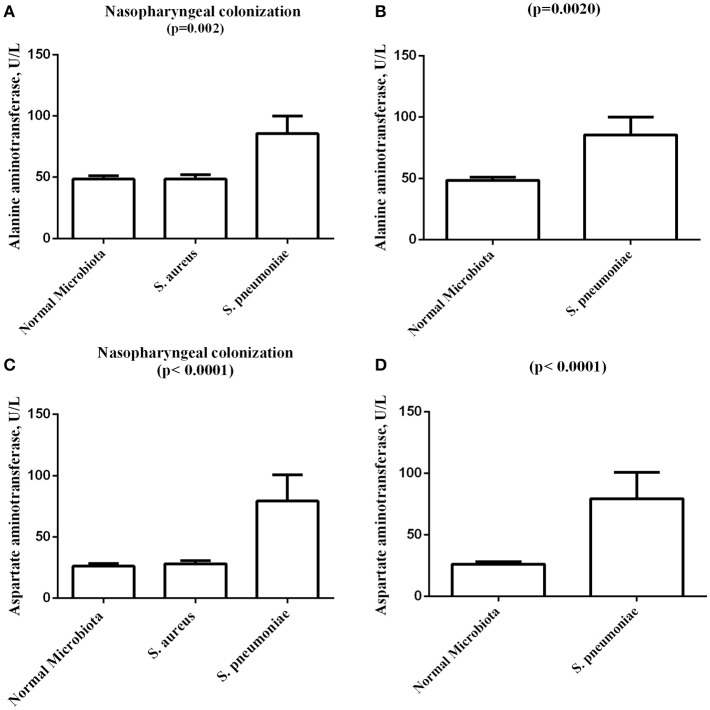
**Graphical representation of the analysis of sickle cell anemia patients nasopharyngeal colonization by normal microbiota, Staphylococcus aureus, and Streptococcus pneumoniae and its association with biochemical variables. (A)** Comparison of alanine aminotransferase levels among normal Microbiota, *S. aureus*, and *S. pneumoniae* nasopharyngeal colonization. **(B)** Comparison of alanine aminotransferase levels between normal Microbiota and *S. pneumoniae* nasopharyngeal colonization. **(C)** Comparison of aspartate aminotransferase levels among normal Microbiota, *S. aureus*, and *S. pneumoniae* nasopharyngeal colonization. **(D)** Comparison of aspartate aminotransferase levels between normal Microbiota and *S. pneumoniae* nasopharyngeal colonization. *S. aureus, Staphylococcus aureus; S. pneumonia, Streptococcus pneumoniae*.

Statistical analysis of alanine transaminase (ALT)-values (U/L) revealed significant differences with a mean and standard deviation of 48.42 ± 22.19 for subjects colonized by normal microbiota (69/143); 48.38 ± 28.55 for individuals colonized by *S. aureus* (64/143); and 85.50 ± 29.22 for individuals colonized by *S. pneumoniae* (8/143) (*p* = 0.02). The analysis also revealed significant differences when comparing ALT-values in individuals colonized by normal microbiota and individuals colonized by *S. pneumoniae* (*p* = 0.002) (Figure 2).

Statistical analysis of aspartate transaminase (AST) values (U/L) revealed significant differences with a mean and standard deviation of 26.21 ± 16.76 for subjects colonized by normal microbiota (693/143); 28.02 ± 21.87 for individuals colonized by *S. aureus* (64/141); and 79.25 ± 43.15 for individuals colonized by *S. pneumoniae* (8/143) (*p* < 0.0001). The analysis also revealed significant differences when comparing AST values in individuals colonized by normal microbiota and individuals colonized by *S. pneumoniae* (*p* < 0.0001) (Figure 2).

The multivariate analysis adjusted for age and sex found that infection was independently associated to oropharynx colonization (*P* = 0.003; *OR* = 4.9; 95% IC 1.7–14.0) and leukocyte count (*P* = 0.0046; *OR* = 8.8; 95% IC 1.9–39.9) (Table 5), and that pneumonia was independently associated to hemoglobin profile (*P* = 0.006; *OR* = 4.3; 95% IC 1.5–12.5) (Table 6).

**Table 5:** **Multivariate analyses models in sickle cell disease patients with infection as the dependent variable**.

**Variables**	**Coefficient**	**Standard error**	**Wald test**	***P*-value**	**Odds ratio**	**95% Confidence limits**
Oropharynx colonization	**1.5902**	**0.5374**	**8.7557**	**0.0031**	**4.9048**	**1.7107–14.0628**
Neutrophils	−1.5627	0.7895	3.9177	0.478	0.2096	0.446**–**0.9848
Leukocytes	**2.1797**	**0.7687**	**8.0398**	**0.0046**	**8.8439**	**1.9602–39.9025**
Platelets	0.0416	0.5254	0.0063	0.9369	1.0425	0.1554**–**1.2470
Hemoglobin profile	0.6092	0.6239	0.9532	0.3289	1.8389	0.5413**–**6.2467
Age	0.4699	0.5334	0.7763	0.3783	1.5999	0.5625**–**4.5507
Sex	0.6091	0.4652	1.7142	0.1904	1.8388	0.7388**–**4.5764
Reticulocytes	−0.8204	0.5312	2.3854	0.1225	0.4402	0.1554–1.2470

*Bold values represent the statistical significant results*.

**Table 6:** **Multivariate analyses models in sickle cell disease patients with pneumonia as the dependent variable**.

**Variables**	**Coefficient**	**Standard error**	**Wald-test**	***P*-values**	**Odds ratio**	**95% confidence limits**
Neutrophils	−0.1696	0.5890	0.8293	0.7733	0.8440	**0**.2661–2.6772
Platelets	0.5888	0.4361	1.8229	0.1770	1.8017	0.7665–4.2351
Reticulocytes	0.1406	0.4469	0.0990	0.7531	1.1510	0.4793–2.7637
Leukocytes	0.3156	0.6099	0.2677	0.6048	1.3711	0.4149–4.5310
Hemoglobin profile	**1.4699**	**0.5403**	**7.4010**	**0.0065**	**4.3489**	**1.5082**–**12.5398**
Oropharynx colonization	0.3235	0.4269	0.5741	0.4486	1.3819	0.5985–3.1906
Neutrophils	−0.1310	0.5862	0.0500	0.8231	0.8772	0.2781–2.7671
Platelets	0.5964	0.4360	1.8713	0.1713	1.8156	0.7725–4.2670
Reticulocytes	0.1431	0.4450	0.1034	0.7478	1.1538	0.4823–2.7603
Leukocytes	0.3091	0.6092	0.2574	0.6119	1.3622	0.4127–4.4961
Hemoglobin profile	**1.4423**	**0.4116**	**7.0774**	**0.0078**	**4.2303**	**1.4618–12.2418**
Nasopharynx colonization	−0.0240	0.4528	0.0034	0.9535	0.9763	0.4357–2.1875

*Bold values represent the statistical significant results*.

## Discussion

The SCD has a high prevalence worldwide with clinical variability and a variety of factors modulating the disease phenotype; these factors make understanding patient characteristics fundamental to improving patient care and to modifying the natural history of the disease (Quinn et al., 2004; Williams et al., 2009).

Infections caused by *S. pneumoniae* are a major cause of morbidity and mortality in children worldwide, particularly in children under 5 years old, and are frequent in individuals with chronic cardiopulmonary diseases and in elderly and immunocompromised individuals (Hausdorff et al., 2000). Similarly, the incidence of *S. aureus* infections are increasing and are now considered a public health problem in the United States of America (Denno et al., 2002; Bogaert et al., 2004b; Cardozo et al., 2006). Although an early onset of prophylaxis with Penicillin and a proper treatment of the infections have increased the overall survival in childhood (Iughetti et al., 2016). Given the high susceptibility to pneumococcal infection, children with SCD are routinely immunized with pneumococcal vaccines and administered prophylactic antibiotics alongside (Mitchell and Mitchell, 2014).

In the current study, we observed nasopharyngeal/oropharynx colonization by *S. pneumoniae* in 11.2% of SCD patients and by *S. aureus* in 56.7% of SCD patients. Steele et al. (1996) observed nasopharyngeal colonization by *S. pneumonia* in 33% of children with SCD who were under two years of age; in 10% of children 2–5 years of age; and in 6% of children 5 years of age. These results were similar to those described by Norris et al. (1996). Fonseca et al. (2005) reported a prevalence of 13.3% for nasopharyngeal colonization by *S. pneumoniae* among Brazilian children with SCD in São Paulo. We emphasize that none of the studies cited above included a description of colonization by other pathogens.

Results obtained in this study allow us to hypothesize that a reduction in nasopharyngeal colonization most likely occurs secondarily to proper use of pneumococcal vaccines suggesting the likely effectiveness of vaccination coverage for serotypes that cause invasive disease. In addition, the *S. aureus* colonization results suggest a possible role of nasopharyngeal colonization by this microorganism in SCD in the children investigated in this study. A similar role has been was suggested by Lee et al. (2009) who studied the epidemiology of and risk factors for colonization with *S. aureus* in children in Massachusetts (USA) and concluded that colonization by methicillin-resistant *S. aureus* remained stable among children from 2003 to 2004 and from 2006 to 2007 despite the widespread use of the pneumococcal conjugate vaccine. Nasal colonization varied with age and was inversely proportional to the recent use of antibiotics.

The profile of pneumococcal strain sensitivity described in the present study revealed no increase in resistance to penicillin compared to data obtained in previous studies in Brazil that included invasive and colonizing strains (Baynes et al., 1986; Moran et al., 2005; Marchese et al., 2011). However, the pneumococcal penicillin sensitivity results presented in this study corroborate those reported by Fonseca et al. (2005) who analyzed 98 Brazilian children from São Paulo and suggested that penicillin remains safe for both prophylaxis and treatment in children with SCD. The authors reported high pneumococcal resistance to clotrimoxazol (sulfamethoxazole/trimethoprim (SMX/TRP) combination) that was 64%. These results suggest that this antibiotic should be used with caution for the treatment of *S. pneumoniae* infections in patients similar to those included in this study.

Most of the identified strains of *S. pneumoniae* were from serotypes that cause invasive diseases in Brazil (Berezin et al., 2002; Mantese et al., 2003). In the present study, we observed the presence of serotypes that dominate in reports of infections in children worldwide (i.e., 6, 14, 19, 23) (Sniadack et al., 1995) with the exception that we did not identify serotype 6. Marchese et al. (2011) evaluated blood cultures from children up to 5 years of age in Italy who were hospitalized for community-acquired pneumonia and identified serotypes 19A and 14 more frequently than other serotypes corroborating the results reported in the present study. Brandileone et al. (1998) reported that infections caused by serotypes 1 and 5 are common in our country; however, in the present study, these serotypes were not isolated corroborating the results reported by Fonseca et al. (2005) and comments made by Berezin et al. (2002) who considered these serotypes significant in the context of nasopharyngeal colonization.

Chemical and hematological measurements are important for monitoring the clinical course of disease in patients with SCD. Children with HbSS exhibited significant differences in almost all analyses when compared to the HbSC group corroborating the results presented by Seixas et al. (2010) who studied children with SCD from the same population. These results also confirm existing data in the literature related to the clinical severity of HbSS compared to other types of SCD (Steinberg and Rodgers, 2001).

Among the biochemical markers analyzed in this study, those associated with inflammation, haemolysis, infection, and oxidative stress were highlighted with an emphasis on patients who exhibited serum ferritin concentrations above the mean values established for the group (≥320.16). Because this protein increases during inflammatory and infectious processes, an accumulation of iron may occur in these patients (Rogers, 1996). SCD is characterized by a proinflammatory state with the presence of abnormal endothelial activation (Belcher et al., 2014). At baseline, ferritin is an important protein for maintaining iron stores in the body. However, during the inflammatory processes, ferritin is regulated by hepcidin and interleukin-6 (IL-6) levels. Other proinflammatory cytokines, such as IL-1β and tumor necrosis factor alpha (TNF-α), also indirectly induce the synthesis of ferritin because they increase the incorporation of iron by hepatocytes (Baynes et al., 1986; Jurado, 1997). The association of a chronic inflammatory state with the observation of increased ferritin values in SCD patients colonized by microorganisms can serve as a marker of clinical severity.

It is known that encapsulated bacteria, in particular *S. pneumoniae*, have a polysaccharide capsule that impedes binding of complement or prevents complement assembled on the cell wall from interacting with macrophage receptors (Bohnsack and Brown, 1986). On the other hand, macrophages engulfing the abnormally sickle shaped cells may become “blocked,” impairing their phagocytosis of other particles, which may contribute to the infection prolongation (Booth et al., 2010). McCavit et al. (2011) described seven invasive pneumococcal diseases due to non-vaccinal serotypes on SCD patients after the pneumococcal 7-valent conjugate vaccine. These facts allow us to suggest that both inflammation and colonization processes are responsible for the abnormal ferritin levels found in our patients.

The results presented in this study demonstrate that patients whose nasopharynx is colonized by *S. pneumoniae* exhibit high levels of AST, ALT, and ferritin. These associations demonstrate that routine biochemical evaluations and assessments of nasopharynx colonization may be important when monitoring the clinical course of SCD patients. The significant differences observed in this study suggest that the colonization by microorganisms is involved in the modulation of inflammatory events in these patients; the presence of pathogens may increase reactive oxidative species maintaining the inflammatory state and increasing disease severity. Unfortunately, we did not compare our findings with previous reports because no similar data were available in the literature.

Studies of the colonization of the nasopharynx and oropharynx by microorganisms in children with SCD can provide important information for public health programs, avoiding severe infections. However, the finding of altered levels of molecules associated with hemolysis and inflammation among SCD individuals with nasopharynx and oropharynx colonization emphasizes the need to elucidate the mechanisms that may determine the development of an invasive infection disease, since the influence of colonization on therapeutic and vaccine effectiveness are poorly understood. Moreover, it is important to clarify the mechanisms by which the biomarkers involved are associated with the presence of these microorganisms and inflammatory processes among SCD patients. This type of information would improve our understanding of these processes and the clinical prognosis in these patients.

## Author contributions

LR and MdSG conceived the study; LR, MC, VN, MSdS, TB, EA, JR, and MdSG designed the study protocols; LR, MC, VN, and MdSG performed the clinical assessments; LR, MC, VN, MSdS, TB, EA, JR, and MdSG performed data analysis and interpreted the data; LR, CD, RS, and MdSG drafted the manuscript; LR, MC, TB, EA, JP, CD, RS, and MdSG critically revised the manuscript for intellectual content. All authors read and approved the final manuscript. MdSG is the guarantor of the paper.

## Funding

This work and was supported by grants from the Brazilian National Council of Research (CNPq) (311888/2013-5) (MdSG); the Foundation Research and Extension of Bahia (FAPESB) (3626/2013, 1431040053063, and 9073/2007) (MdSG); and PPSUS/FAPESB (020/2013 EFP-00007295), (MdSG); the Instituto Nacional de Ciência e Tecnologia do Sangue (CNPq) (Coordinated by S.T.O.S.), and MCD/CNPq/MS-SCTIE-DECIT (409800/2006-6), (MdSG). Sponsors of this study are public or nonprofit organizations that support science in general. They had no role in gathering analyzing, or interpreting the data.

### Conflict of interest statement

The authors declare that the research was conducted in the absence of any commercial or financial relationships that could be construed as a potential conflict of interest.
